# *Mycobacterium chimaera*: a report of 2 new cases and literature review

**DOI:** 10.1007/s00414-021-02630-y

**Published:** 2021-06-29

**Authors:** Alice Natanti, Marco Palpacelli, Marco Valsecchi, Adriano Tagliabracci, Mauro Pesaresi

**Affiliations:** 1 Section of Legal Medicine, Department of Excellence SBSP—Biomedical Sciences and Public Health, Università Politecnica delle Marche of Ancona, Conca 71, Street, Ancona, Italy; 2 SOD of Legal Medicine, Azienda Ospedaliero Universitaria Ospedali Riuniti “Umberto I G M Lancisi G Salesi”, Ancona, Italy

**Keywords:** *Mycobacterium chimaera*, Granuloma, Cardiovascular surgery, Heater-cooler units, Healthcare-associated infection

## Abstract

*Mycobacterium chimaera* is a non-tuberculous mycobacterium, member of the *Mycobacterium avium* complex (MAC), which has become a global public health concern due to infection following cardiac surgery performed with contaminated heater-cooler units. *M. chimaera* infection is characterized by a long latency, non-specific signs and symptoms and high mortality rates. Thus, the diagnosis is still challenging both for forensic pathologists and for clinicians. Clinical manifestations of *M. chimaera* infection include endocarditis, hepatitis, nephritis, encephalitis and chorioretinitis. A constant histopathologic finding is the presence of non-caseating granulomas, with multinucleated giant cells and histiocytes. Hereby, we present two cases of fatal disseminated *M. chimaera* infection following aortic valve surgery reporting clinical history and post-mortem findings. Further, we provide a brief overview of the literature with a special focus on histopathological characteristics of *M. chimaera* infection. The aim of this article is to provide a complete synopsis of histopathological characteristics useful for forensic pathologists.

## Introduction

*Mycobacterium chimaera* is a non-tuberculous mycobacterium first identified in 2004 [1], which is part of the *Mycobacterium Avium* complex (MAC). It is an opportunistic pathogen responsible for respiratory infection mainly in immunocompromised subjects and in patients with underlying respiratory diseases such as cystic fibrosis [2].

During last years, *M. chimaera* has become a global public health concern due to infection following cardiac surgery because of contaminated devices, called heater-cooler units (HCU), used to regulate blood temperature during extracorporeal circulation [3]. It seems that *M. chimaera* forms biofilms in heater-cooler unit water tanks of contaminated devices and then spreads through airborne transmission [4]. In 2013, Achermann et al. described the first cases of prosthetic valve endocarditis and bloodstream infection due to *M. chimaera* [5], while an outbreak of *M. chimaera* infections has been reported in 2015 among European patients who underwent open-chest surgery performed using a specific brand of heater-cooler devices (HCD) [6–8]. The first case of *M. chimaera* infection in Italy was described in December 2016, in a woman with a history of cardiac surgery who developed disseminated infection and vertebral osteomyelitis [9].

*M. chimaera* infection is characterized by a long latency between infection and onset of symptoms which varies from 1 to 6 years. Signs, symptoms and laboratory features are often non-specific and include low-grade fever, persistent cough, muscle pain, abdominal pain, pus at the surgical site and vomiting [3]. If not promptly diagnosed and properly treated, *M. chimaera* infections may become life-threatening [10]. Currently, no standardized treatment for *M. chimaera* infection exists [11]. Thus, antibiotic therapy should be guided by the results of a drug susceptibility test performed in a reference center for mycobacterial pathogens [3], and revision surgery has to be evaluated case-by-case.

Here, we present two cases of fatal disseminated *M. chimaera* infection, following valve replacement surgery performed with contaminated heater-cooler units, with a special focus on histopathological aspects.

### Case history

#### Case 1

A 74-year-old male underwent bioprosthetic aortic valve replacement for severe regurgitation on December 2015. In February 2018, the patient was submitted to prostate resection. The results of histological analysis on biopsy samples revealed acute and chronic granulomatous inflammation.

In May 2018, the man was admitted to the hospital because of persistent left hemithorax pain and left abdominal pain accompanied by splenomegaly and an episode of dysarthria. Brain MRI with contrast showed bilateral multiple ischemic lesions suggestive of microangiopathic changes. At transoesophageal echocardiogram, no prosthetic vegetation nor abscesses were detected, and blood cultures were negative. On July 2018, a positron-emission tomography and computed tomography (PET-CT) scan showed increased peri-prosthetic metabolic activity. Thus, the man underwent serological analysis and mycobacterial culture that revealed the presence of *M. chimaera* in blood, urine, feces and bone marrow. Antibiotic therapy with clarithromycin, rifabutin and ethambutol was prescribed. Despite targeted drug therapy, the patient died on May 2019, at the age of 77, due to progressive multiple organ failure.

#### Case 2

A 66-year-old female underwent bioprosthetic aortic valve replacement associated with aortic root vascular replacement on May 2015.

On March 2017, the woman was admitted to the hospital for persistent fever, somnolence, asthenia, night sweats, hepatomegaly and splenomegaly. Blood cultures were negative while PET-CT scan revealed liver increased metabolic activity. Liver biopsy was performed, and tissue culture showed the presence of *M. chimaera*. After 102 days of hospitalization, she was discharged with the prescription of levofloxacin, rifampicin and clarithromycin.

On August 2017, the woman was hospitalized again due to fever, asthenia and ascites resistant to therapy. Blood cultures demonstrated non-tuberculous mycobacteria, and a new PET-CT scan indicated increased metabolism around the aortic prosthesis. Once again, no signs of endocarditis were pointed out. Antibiotic therapy was modified with the introduction of rifabutin and clofazimine instead of levofloxacin and rifampicin. In January 2019, the hospital where the woman underwent the aortic valve replacement sent alerts regarding the possible risk of *M. chimaera* post-surgical infections.

A brain MRI performed on March 2019 showed bilateral subacute ischemic lesions caused by septic embolization. On the same occasion, splenic infarctions were seen at abdomen CT scan. The patient died on August 2019, at the age of 70.

## Materials and methods

### Post-mortem examination

Post-mortem examination was performed 12 days (Case 1) and 2 days (Case 2) after the death and included the revision of clinical records requested to the hospital and the sampling of tissues for histological analysis.

### Histology

Tissue samples were fixed in formalin, dried, clarified, paraffin embedded and cut with a microtome in order to obtain Sects. 6–8 μm thick. Histological sections were stained with Hematoxylin and Eosin (H&E) or Ziehl–Neelsen. Finally, the slides were observed with an optical microscope. Photomicrographs were taken using a PrimoStar iLED microscope (Zeiss, Germany).

### Microbiology

Tissues were mechanically homogenized in phosphate-buffered saline (PBS) using a TissueLyser II (Qiagen, Germany). The homogenates were serially diluted and subsequently decontaminated from other environmental microorganism using N-acetyl-L-cysteine sodium hydroxide (NALC- NaOH).

MGIT 960 microbiology system (Becton Dickinson and Co., Sparks, MD) and Middlebrook 7H11 agar for liquid and Lowenstein-Jensen agar for solid were used respectively. Plates were monitored weekly for growth. The presence of mycobacteria on 7H11 media plates as well as in liquid media was confirmed by Ziehl–Neelsen stain. Species identification of the mycobacterium was made with probes from AccuProbe-Hologic, San Diego, CA, USA.

For the identification of the *M. chimaera* species, a genetic analysis was performed with the GenoType NTM-DR VER 1.0 Kit, Hain Lifescience Arnika.

### Review of the literature

A literature search was first conducted using the Medline Database (PubMed.gov; US National Library of Medicine-National Institute of Health) and free text protocols (i.e. “*Mycobacterium chimaera*”), individually combined through the Boolean operator “AND”. Further studies were identified by reviewing the reference lists of the papers previously found. The search resulted in more than 160 articles, but our study included only articles that contain references to histologic findings (e.g. granuloma). Data are summarized in Table 1. Approximately 7 articles reported histological examination, and their texts were fully analyzed.

**Table 1:** Cases of *Mycobacterium chimaera* infection following cardiac surgery

Authors	Age (y)	Sex	Surgery or other	Latency (month)	Other tissue involvement	Heart	Kidney	Liver	Brain	Lungs	Histopathological findings	Death
Trautman C. et al	63	F	AVR	72	Anemia	Prosthetic valve vegetations, aortic root abscess	Renal impairment	nd	nd	nd	Bone marrow granuloma, granulomatous interstitial nephritis	No
Watanabe R. et al	61	M	Seronegative rheumatoid arthritis	/	Tenosynovitis	nd	nd	nd	nd	nd	Inflammatory cell infiltration and multinucleated giant cells in synovial tissue	No
Böni C. et al	51	M	Open-heart surgery	16	Progressive choroiditis	Endocarditis and/or aortic graft infection	nd	nd	nd	nd	nd	Yes
	64	M		39	Choroidal lesions						nd	No
	49	M		41	Progressive choroiditis						nd	No
	61	M		21	Progressive choroiditis						nd	Yes
	63	M		22	Progressive choroiditis						nd	Yes
	64	M		21	Choroidal lesions						nd	No
	66	M		36	Progressive choroiditis						nd	No
	50	M		26	Choroidal lesions						nd	No
	58	M		25	Choroidal lesions						nd	No
Sandrine A. et al	51	M	Composite graft replacement	16	Fever, uveitis, vitritis and choroidal lesions, splenomegaly, pancytopenia	nd	nd	Hepatitis	nd	Pneumonitis	nd	Yes
	65	M	Mitral valve reconstruction	39	Uveitis, vitritis and choroidal lesions, splenomegaly, pancytopenia	Endocarditis with cardiac insufficiency	Renal impairment	Hepatitis	nd	nd	nd	No
	49	M	AVR	41	Arthritis, choroidal lesions, splenomegaly, pancytopenia	Endocarditis with cardiac insufficiency	nd	Hepatitis	nd	nd	nd	No
	61	M	Aortic root and arch replacement	21	Splenomegaly, bicytopenia, vertebral osteomyelitis, choroiditis	nd	Renal failure	Hepatitis	nd	nd	nd	Yes
	63	M	Aortic root and arch replacement	22	Splenomegaly, bicytopenia, choroiditis and anterior uveitis	nd	Renal impairment	Hepatitis	nd	nd	Granulomatous inflammation of choroid, kidneys and brain	Yes
Overton K. et al	83	F	AVR	13	Pancytopenia	Fludeoxyglucose (FDG) avidity around the prosthetic aortic valve	Renal impairment	Liver function test derangement	nd	nd	nd	Yes
	40	M	AVR	23		Severe peri-prosthetic aortic valve regurgitation	Renal impairment	Liver function test derangement	nd	Pneumonia	Reactive changes in bone marrow, renal suppurative granuloma	No
	79	M	AVR + CABG	21	Thrombocytopenia	Large vegetation on the prosthetic valve, aortic root abscess	Renal impairment	Liver function test derangement	nd	nd	nd	No
	63	M	AVR	21	Pancytopenia	nd	nd	Liver function test derangement	nd	nd	Bone marrow with multiple non-caseating granulomas	No
Lau D. et al	60	F	AVR + MVR	15	Pancytopenia, lymphadenopathy, choroidal nodules	nd	nd	nd	nd	nd	Partially necrotizing granulomatous inflammation in liver, kidneys, heart, brain, lungs, spleen, pancreas and thyroid	Yes
	75	M	AVR + root replacement	15	Pancytopenia, lymphadenopathy, choroidal nodules	nd	nd	nd	nd	nd		Yes
	73	F	AVR	12	Choroidal lesions	nd	nd	nd	nd	nd		Yes
Tan N. et al	66	M	Ascending aortic aneurysm prosthetic graft repair	26	Bilateral chorioretinitis	Fludeoxyglucose (FDG) avidity between the ascending aortic graft and the anterior mediastinum	nd	nd	nd	nd	Bone marrow with non-caseating granulomas	No
	74	M	AVR + aortic root repair	26	nd	nd	nd	nd	nd	nd		Yes
	57	M	AVR	16	Splenomegaly, pancytopenia, bilateral chorioretinal lesions	Endocarditis	nd	nd	nd	Pulmonary infiltrates		Yes
Cai Y. et al	63	F	AVR	60	Anemia	Aortic root abscess, previous mitral valve endocarditis	Acute kidney dysfunction	nd	nd	nd	Bone marrow granulomas and amyloidosis, interstitial nephritis with one granuloma	No
Shafizadeh N. et al	56	M	AVR + aortic root repair	14	Pancytopenia	nd	Acute kidney injury		nd	nd	Bone marrow granulomas, sinusoidal granulomas with architectural changes of venous outflow obstruction	Yes
	69	M	AVR + MVR	22	Pancytopenia, bone marrow ill-defined granulomas	Vegetation on both prosthetic valves	nd	Hepatitis C, liver function test derangement, hepatomegaly	nd	nd	Bone marrow granulomas, macrovesicular steatosis, sinusoidal granulomas with architectural changes of venous outflow obstruction	Yes
	76	M	AVR	14	Thrombocytopenia	nd	nd	Liver function test derangement	nd	nd	Sinusoidal granulomas with architectural changes of venous outflow obstruction	Yes
	70	M	AVR + aortic root replacement	21	nd	Vegetation on aortic valve	nd	nd	nd	nd	Granulomatous inflammation of bone marrow, kidneys and liver, sinusoidal granulomas with architectural changes of venous outflow obstruction	Yes
	81	F	AVR	20	nd	nd	nd	Liver function test derangement	nd	nd	Sinusoidal granulomas with architectural changes of venous outflow obstruction	No
	58	F	AVR + aortic root replacement	29	Leukopenia, anemia	nd	nd	Liver function test derangement	nd	nd	Sinusoidal granulomas with architectural changes of venous outflow obstruction	Yes
	62	M	AVR	26	nd	nd	nd	Liver function test derangement	nd	nd	Sinusoidal granulomas with architectural changes of venous outflow obstruction	No
Sax H. et al	58	M	MVR	33	Splenomegaly, pancytopenia	Endocarditis	Renal impairment	Hepatitis	nd	nd	Granulomatous nephritis and hepatitis	Yes
	51	M	Composite graft for aortic dissection	17	Splenomegaly, pancytopenia, ocular emboli	nd	nd	Hepatitis	nd		Granulomatous myocarditis, nephritis and pneumonitis	Yes
	64	M	Mitral valve reconstruction	42	Splenomegaly, pancytopenia, ocular emboli, wrist arthritis	Endocarditis	Renal impairment	Hepatitis	nd	nd	Granulomatous endocarditis, osteomyelitis	No
	49	M	AVR	40	Splenomegaly, pancytopenia, ocular emboli, pacemaker pocket infection	Endocarditis	nd	Hepatitis	nd	nd	Granulomatous hepatitis, myositis	No
	61	M	Aortic root and arch replacement	19	Splenomegaly, ocular emboli	nd	nd	nd	nd	nd	Granulomatous vertebral and sternal osteomyelitis	No
	63	M	Aortic root and arch replacement	20	Splenomegaly, multifocal choroiditis	nd	Renal failure	Hepatitis	nd	nd	Granulomatous interstitial nephritis	No
Kohler P. et al	58	M	Mitral valve reconstruction	24	Anemia, lymphocytopenia, thrombocytopenia, splenomegaly	Cardiac insufficiency	nd	nd	nd	nd	Necrotizing endocarditis	Yes
	51	M	Composite aortic graft replacement	14	Anemia, lymphocytopenia, thrombocytopenia, splenomegaly	nd	nd	nd	nd	nd	Granulomatous myocarditis, nephritis and hepatitis, granulomatous lesions in brain	Yes
	64	M	Mitral valve reconstruction	26	Anemia, lymphocytopenia, thrombocytopenia, splenomegaly	nd	nd	nd	nd	nd	Granulomatous endocarditis and osteomyelitis	No
	49	M	AVR	40	Anemia, lymphocytopenia, thrombocytopenia, splenomegaly	Cardiac insufficiency	nd	nd	nd	nd	Granulomatous pectoral myositis and hepatitis	No
	61	M	Aortic root and arch replacement	17	Anemia, lymphocytopenia, thrombocytopenia, splenomegaly	nd	Nephritis	nd	nd	nd	Granulomatous endocarditis, osteomyelitis and granulomatous lesions in brain	Yes
	63	M	Aortic root and arch replacement	21	Anemia, lymphocytopenia, thrombocytopenia, splenomegaly, osteomyelitis	nd	nd	nd	nd	nd	Granulomatous periaortal tissue and granulomatous nephritis	Yes
	76	M	AVR	22	Anemia, lymphocytopenia, thrombocytopenia, splenomegaly, myositis	Cardiac insufficiency	nd	nd	nd	nd	nd	No
	36	F	Mitral valve reconstruction	5	Anemia, lymphocytopenia, thrombocytopenia, myositis	Cardiac insufficiency	nd	nd	nd	nd	Granulomatous endocarditis	Yes
	74	M	AVR + CABG	10	Anemia, lymphocytopenia, thrombocytopenia	Clinical signs of endocarditis	nd	nd	nd	nd	Granulomatous osteomyelitis and hepatitis, bone marrow with non-necrotizing granulomas	No
	1	M	Aortic arch reconstruction	13	Anemia, lymphocytopenia, thrombocytopenia, splenomegaly	Cardiac insufficiency	nd	nd	nd	nd	nd	No
Asadi T. et al	62	M	Aortic root, ascending aorta and aortic arch replacement	16	Mild anemia, choroid lesions, vertebral osteomyelitis, walled abscess in the left psoas muscle	nd	nd	Liver function test derangement	nd	nd	Non-necrotizing granulomatous hepatitis	No
	65	M	AVR + aortic, hemashield graft placement	36	Pancytopenia, bone marrow non-caseating granulomas	Aortic root abscess	Renal failure	Liver function test derangement	nd	nd	Bone marrow non-caseating granulomas	No
Achermann Y. et al	58	M	AVR + MVR	12	nd	Severe mitral and aortic insufficiency	nd	nd	nd	Respiratory distress	Granulomatous inflammation of kidneys and liver, acute necrotizing mycobacterial endocarditis	Yes
	51	M	Composite aortic graft replacement	16	Splenomegaly, pancytopenia	Prosthetic valve endocarditis	Progressive renal insufficiency	Liver function test derangement	nd	nd	Acute and chronic granulomatous inflammation of kidneys, liver and spleen	Yes
Rosero C. I. et al	66	M	Cough, low-grade fever and weight loss, lung mass treated with partial left lung lobectomy	nd	nd	nd	nd	nd	nd	nd	Necrotizing granuloma with acid fast bacilli in left lung	No
Sebastian Haller S. et al	80	M	AVR	10	nd	Endocarditis	nd	nd	nd	nd	nd	No
	75	M	CABG	60	Spondylodiscitis	nd	nd	nd	nd	nd	nd	No
	65	M	AVR	36	nd	Valvular aortic endocarditis, paravalvular leak and abscess	nd	nd	nd	nd	nd	Yes
	67	M	AVR + CABG	48	nd	Paravalvular abscess	nd	nd	nd	nd	nd	No
	53	M	AVR	36	nd	Endocarditis	nd	nd	Cerebral abscesses	nd	nd	No
Sacco K. A. et al	63	F	AVR	12	Leukopenia, thrombocytopenia	Prosthetic valve endocarditis and root abscess	Renal granulomas	nd	nd	nd	Bone marrow with non-specific granuloma	No
Joseph Butterworth J. et al	72	M	AVR	28	Pancytopenia, splenomegaly	nd	nd	nd	nd	nd	Bone marrow with non-necrotic microgranulomas	No

*AVR* aortic valve replacement, *MVR* mitral valve replacement, *CABG* coronary artery bypass grafting, *nd* non detected

## Results

### Post-mortem examination

#### Case 1

The victim is a 77-year-old Caucasian male in quite good overall physical conditions.

At external examination, cadaveric temperature was lower than the environmental one consistent with the stay in mortuary refrigerator; lividity was reddish, scarce, unbleached on thumb pressure and located at the posterior regions of the body; rigidity appeared completely resolved in the whole body. Mucosal ulcers were observed into the oral cavity, and a linear 27-cm-long scar was seen in the sternal region.

At gross examination, the brain was affected by mild atrophy, oedema and encephalomalacia, particularly in right frontal, parietal and occipital lobes, left temporal and parietal lobes and in the cerebellum. Down the midclavicular line, fractures from the first to the fourth left costa were seen. Bilateral hydrothorax (500 ml in the right pleural space and 600 ml in the left pleural space) and hemoperitoneum (600 ml) were observed, too. The heart was enlarged (750 g) and characterized by adherent pericardium, slight left ventricular hypertrophy and whitish myocardial areas. The aortic bio-prosthesis was correctly located and without signs of endocarditis. Left anterior descending artery showed atherosclerosis. Lungs appeared expanded, weighting respectively 560 g the right lung and 540 g the left one. Splenomegaly (2240 g) and multiple whitish infarction areas were detected in the spleen. Other findings consisted in hepatomegaly (2350 g), bilateral kidney atrophy associated with thinner renal cortex and a sclerotic and ectatic abdominal aorta.

#### Case 2

The victim is a 70-year-old Caucasian female in poor overall physical conditions.

At external examination, cadaveric temperature was consistent with the stay in a mortuary refrigerator; lividity was red- purplish, scarce, unbleached on thumb pressure and located at the posterior regions of the body; rigidity was easily overcome. As distinguishing features, multiple linear scars were observed in the sternal region, right hypochondrium, down the linea alba and in the right iliac region. Red-purplish bruises were detected in the posterior and lateral regions of the neck, in the clavicular region bilaterally and in both arms.

At the section, brain oedema was observed. Pleural spaces as well as peritoneal cavity showed spread adhesions and severe ascites (4000 ml). The heart was slightly enlarged with mild left ventricular hypertrophy. Aortic valve prosthesis and vascular aortic prosthesis were correctly located without signs of endocarditis. Coronary arteries were characterized by non-obstructive atherosclerotic plaques. Lungs were thicker and with an increased blood amount. The spleen showed a slight increased volume (320 g) and an infarction at its lower pole. Mild hepatomegaly (1400 g), thinner renal cortex and peripancreatic fat necrosis were also observed. The abdominal aorta was atherosclerotic and ectatic.

## Histology

### Case 1

**Fig. 1:**
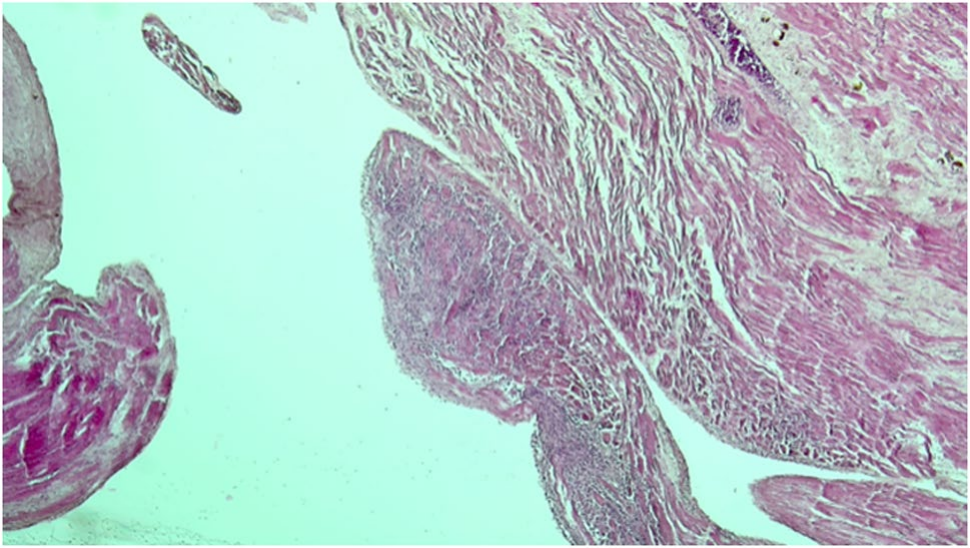
Photomicrograph of myocardium showing a granuloma (Hematoxylin and Eosin stain, × 10)

**Fig. 2:**
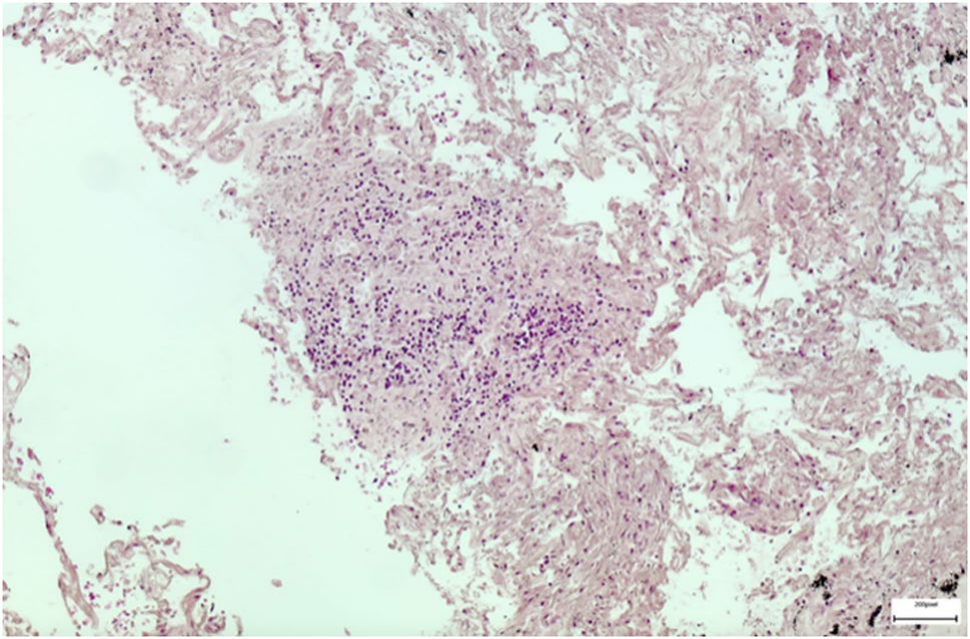
Photomicrograph of right lung showing a granuloma (Hematoxylin and Eosin stain, × 10)

Brain samples showed cortical-subcortical malacic areas associated with increased glial component, infiltrates of granulocytes and hemosiderin deposition both in the hemispheres and in the brain stem. Perivascular and pericellular optically empty spaces and petechial hemorrhages were also observed. The histopathological examination of cardiac tissue revealed a slight increase in the content of perivascular fibrous tissue, areas of replacement fibrosis and severe and widespread granulomatous lesions consisting of histiocytes, multinucleated giant cells and plasma cells (Fig. 1). Lungs showed anthracosis and airspace enlargement with fragmented alveolar walls alternating with collapsed parenchymal areas. Well-formed granulomas were predominantly detected in the right lung (Fig. 2). Portal inflammation with lymphocytic infiltration, lobular necroinflammatory activity and fibrosis were observed in liver. Arterionephrosclerosis with medial thickening of medium-sized arteries, glomerulosclerosis and tubulointerstitial fibrosis were also noted.

### Case 2

**Fig. 3:**
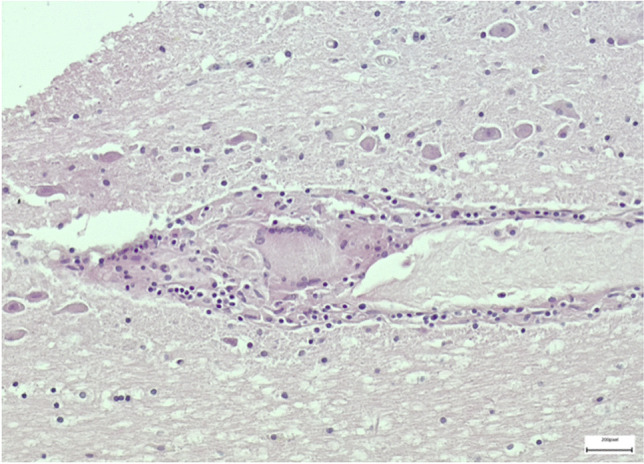
Photomicrograph of brain stem showing a granuloma with the typical Langhans giant cell (Hematoxylin and Eosin stain, × 20)

**Fig. 4:**
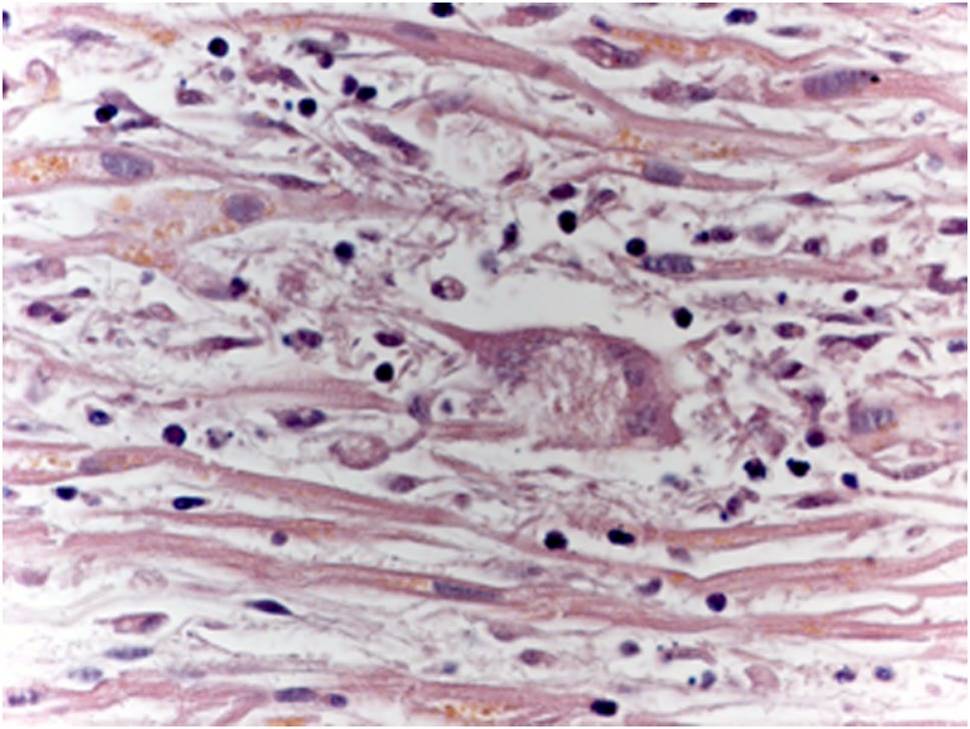
Photomicrograph of myocardium showing a Langhans giant cell, lymphocytes and plasm cells (Hematoxylin and Eosin stain, × 40)

Brain samples showed pericellular optically empty spaces and petechial hemorrhages as well as widespread granulomas consisting of lymphocytes, histiocytes and rare multinucleated giant cells surrounded by a lymphocytic and macrophagic infiltrate (Fig. 3). Granulomatous lesions were identified also in heart myocardial samples (Fig. 4), associated with areas of replacement fibrosis and increased perivascular fibrous tissue. The aortic paravalvular tissue examination revealed multinucleated giant cells and fibrosis. Lungs showed pleural thickening and airspace enlargement with fragmented alveolar walls alternating with collapsed parenchymal areas. Liver samples revealed a microscopic pattern of chronic hepatitis consisting in enlargement of portal tracts, fibrosis, lymphocytic infiltrates and portal-portal fibrous bridging. Arterionephrosclerosis with medial thickening of medium-sized arteries and glomerulosclerosis were also seen.

### Microbiology

#### Case 1

*M. chimaera* was detected post-mortem in patient’s bone marrow, lymph nodes, spleen, brain and liver samples.

#### Case 2

*M. chimaera* was identified post-mortem in patient’s lymph nodes, spleen, brain and peri-prosthetic tissue.

## Discussion

Since 2013, *Mycobacterium chimaera* infections due to specific brands of contaminated heater-cooler units used in cardiac surgery have been concerning public health worldwide.

Many authors have shown that heater-cooler units used to regulate patient’s body temperature during cardiac surgery procedures have been colonized by *Mycobacterium chimaera* [8, 12, 13]. For example, LivaNova Stockert 3T models might have been originally contaminated in German production site [4] even though a contamination during their use cannot be excluded. Since 2014, SORIN Group Deutschland GmbH and Maquet Getinge Group have issued several security alerts finalized to inform about the procedures that have to be adopted in case of specific contaminated units, providing their serial numbers. In particular, the alerts stressed the importance of devices’ cleaning and disinfection, water quality checking and the usefulness of directing the devices’ drain away from the patient. Moreover, the manufacturer recommended to promptly removed from the operating rooms the heater-cooler units suspected to be contaminated [14].

The review of the literature showed that *Mycobacterium chimaera* infections involved patients aged from 12 months to 83 years with a median age of 60.4 years. Regarding the type of surgical intervention, infection followed aortic valve replacement (AVR) alone (*n* = 19) or in combination with aortic root replacement or repair (*n* = 6), mitral valve replacement (MVR) (*n* = 3) or coronary artery bypass grafting (CABG) (*n* = 3). Infection followed also aortic root and arch replacement (*n* = 7), mitral valve reconstruction (*n* = 5), composite graft replacement (*n* = 4), CABG (*n* = 1), MVR (*n* = 1), aortic arch reconstruction or repair (*n* = 2), lung lobectomy (*n* = 1), history of seronegative rheumatoid arthritis treated with methotrexate, tacrolimus and prednisolone (*n* = 1), nd (*n* = 1).

The most common presenting symptoms include fever, night sweats and weight loss [10]. In addition, lymphopenia, thrombocytopenia, anemia, elevated levels of creatinine, transaminases and C-reactive protein are often encountered [19].

The diagnosis could be difficult because signs and symptoms are non-specific, slight and appear generally from 6 weeks to more than 5 years after surgery. It is interesting to note that some patients were misdiagnosed with sarcoidosis after the discovery of granulomatous involvement and initiated on steroid therapy [20].

According to Sax et al. [17], the latency period is long, with a median of 26 months.

Moreover, extracardiac symptoms may precede the cardiac ones, and a cardiac involvement can be detected only at post-mortem examination.

*M. chimaera* infections mortality rate may reach 60% [15], probably due to multiple factors including the risk of reoperative surgery, the long latency of the infection, the intrinsic antibiotic resistance of these slow-growing mycobacteria, the prolonged antibiotic therapy and the infected sites that may be challenging for antimicrobial penetration [19].

Patients could experiment prosthetic valve endocarditis, vascular graft infections and/or bacteraemia with manifestations that can vary from splenomegaly to arthritis, osteomyelitis, bone marrow involvement with subsequent cytopenia, chorioretinitis, lung involvement, hepatitis, nephritis and myocarditis [3].

Especially, the analyzed studies revealed that patients presented signs of involvement of single or multiple organs including endocarditis (*n* = 20), cholestatic hepatitis (*n* = 20), granulomatous nephritis (*n* = 12), cytopenia (*n* = 10), osteomyelitis or other bone lesions (*n* = 9), encephalitis (*n* = 7), chorioretinitis or vasculitis (*n* = 6), aortic valve tissue inflammation (*n* = 6), pneumonitis (*n* = 3), spleen inflammation (*n* = 2), myositis (*n* = 2), uveitis and vitritis (*n* = 1) and inflammatory cell infiltration of synovial tissue (*n* = 1).

As routine blood cultures have a low mycobacterial growth sensitivity, suggested methods for diagnosis are mycobacterial blood cultures, performed multiple times on separate days to maximize their sensitivity, together with molecular diagnostics tools such as polymerase chain reaction (PCR) [3]. The use of molecular probes with 16S rDNA sequencing and rpoB sequencing is essential to identify *M. chimaera* among other members of the MAC [17, 18].

However, *Mycobacterium* species can require 14–21 days of incubation on culture media before their detection. Thus, a thorough histopathological examination of bioptic samples may show a pattern of injury indicative of granulomatous disease, and then it can anticipate the diagnosis.

In fact, the main histologic feature of *M. chimaera* infection is represented by non-caseating granuloma and foamy/swollen macrophages with or without acid-fast bacilli [19].

A granuloma is the result of chronic inflammation and consists of a microscopic aggregation of macrophages transformed into epithelioid cells, surrounded by a collar of lymphocytes and plasma cells. The fusion of epithelioid cells forms the so-called Langhans giant cells with the typical arrangement of nuclei in a horseshoe-shaped pattern near the outer edge of the cell or in cluster at the two poles of the cell [21].

In the examined articles, granulomas involved kidney (*n* = 2), liver (*n* = 3), spleen (*n* = 1), brain (*n* = 1), heart, lung and choroidal tissue.

In our cases, granulomatous lesions were observed respectively in myocardium and lungs (case 1) and in brain and myocardium (case 2), indicating a disseminated infection.

Therefore, maximum effort should be made to obtain biopsy for histologic analysis: the detection of non-caseating granulomas, foamy macrophages or multinucleated giant cells in cardiac tissue and in other tissues should prompt the clinicians to search for a history of open-heart surgery and to set up the most appropriate diagnostic and therapeutic interventions.

In our case 2, a liver biopsy was performed 2 years before the death as a previous PET-CT scan has revealed liver increased metabolic activity. This allowed the diagnosis of *M. chimaera* infection. Nevertheless, the prognosis has been poor anyway probably due to the dissemination of the pathology that had already occurred.

In some cases, the presence of granulomatous inflammation in multiple organs has led to an initial misdiagnosis of sarcoidosis [20, 22] with consequent administration of immunosuppressive therapies which may also have contributed to poor outcomes. However, the presence of extrapulmonary localizations and bone-marrow involvement is infrequent in sarcoidosis and should be properly considered [23]. Hence, it is necessary to stress the importance of a correct differential diagnosis, since the misinterpretation of these cases as sarcoidosis or other immuno-mediated diseases may produce a worse outcome for these patients.

It is also recommended to perform a retinal examination in suspected cases, even without visual symptoms, due to the possibility of detecting rapidly choroidal granulomas that would be suggestive of a disseminated *M. chimaera* infection [24].

The level of awareness of healthcare professionals is currently improved thanks to specific alerts spread by national or regional government agencies. High clinical suspicion for non-tuberculous mycobacteria infection is strongly recommend in case of cardiac prosthetic valve endocarditis, prosthetic vascular graft infection, sternotomy wound infection, mediastinitis and signs of disseminated infection including embolic and immunologic manifestation, in patients who have undergone cardiac surgery requiring heater-cooler units in the 6 years prior the onset of symptoms. Recognition of this pattern of injury can lead to a correct diagnosis so that a suitable antibiotic therapy can be initiated as early as possible in order to reduce morbidity and mortality.

Forensic pathologists need to pay attention to the clinical history of the victim with a thorough examination of clinical records in order to assess the presence of previous cardiopulmonary surgery performed with heater-cooler devices and to ascertain signs and symptoms suggestive of infection. If *M. chimaera* isolation has not been realized when the subject was still alive, post-mortem microbiological investigations have to be carried out. Then, histological analysis of various tissue samples is essential. The detection of granulomatous lesions either localized in only one tissue or spread in different organs could be highly suggestive of mycobacterial infection and, in the same time, could give precious information about the dissemination of the disease.

In conclusion, the management of *M. chimaera* infection is still challenging. Morbidity and mortality are high due to the difficulties related both to diagnosis and to therapy. Forensic pathologists, even if in the absence of a previous diagnosis of *M. chimaera* infection, could easily reach the correct diagnosis based on correlation between clinical history, post-mortem examination and laboratory investigations in which histological analysis plays a fundamental role in order to detect the typical granulomatous lesions.

In Italy, probably there will be an increase in *M. chimaera* infections’ prevalence in the coming years. In fact, until a few years ago, little or nothing was known of this infection related to contaminated heater-cooler units, and the long incubation time of this kind of disease suggests a possible short-term spike in *M. chimaera* infection diagnosis.

Thus, it is essential to increase the level of awareness both among clinicians and among pathologists in order to have skills and tools to face this serious surgical-related infection.

## Data Availability

Data sharing is not applicable to this article as no datasets were generated or analyzed during the current study.
